# President’s Address, Forty-eighth Annual Session of the Medical Association of Georgia, Macon, April 21, 1897

**Published:** 1897-05

**Authors:** Geo. H. Noble

**Affiliations:** Atlanta, Ga., President of the Association


ATLANTA
Medical and Surgical Journal.

VOL. XIV. MAY, 1897. No. 3.

LUTHER B. GRANDY, M.D., MANAGING EDITOR. M. B. HUTCHINS, M.D., BUSINESS MANAGER.

COLLABORATORS:
A. W. CALHOUN, M.D., LL.D., VIRGIL O. HARDON, M.D., FLOYD W. McRAE, M.D.,
A. W. STIRLING, M.D., JOSEPH EVE ALLEN, M.D., AUGUSTA, GA., AND
HENRY R. SLACK, PH.M., M.D., LAGRANGE, GA.

### *ADDRESSES.*

### PRESIDENT'S ADDRESS, FORTY-EIGHTH ANNUAL SESSION OF THE MEDICAL ASSOCIATION OF GEORGIA, MACON, APRIL 21, 1897.

BY GEO. H. NOBLE, M.D., ATLANTA, GA.,
President of the Association.

It is with feelings of deep pleasure and gratitude that I attempt to execute the customary functions of the high office with which you have honored me, and it is hoped that you will bear with patience and much charity my shortcomings in the discharge of the same. I am sure that our Association is to be congratulated that we are assembled again in Georgia's hospitable Central City in such inspiring circumstances. The excellent program, unprecedented in extent, and this splendid attendance are the token and the pledge of continued work that shall promote the welfare of its membership, and that it shall go on performing its high objects of good to the profession and the public.

If by recounting some of the reasons why we should feel an enduring pride in our calling, or by speaking some word of encouragement or suggestion, I may, in however small a measure, promote the welfare of our organization, I shall feel that I have not in a wholly unworthy manner performed the task of making the usual annual address.

An endeavor to trace the beginning of the practice of medicine will lead the inquirer to the Olympian heights, and will give him mythological authority, at least, for asserting its divine origin. Perhaps the first allusion to the medical practitioner is to be found in the history of the Centaurs—creatures described as half man and half beast. Thus we find Chiron described as having the head and body of a man joined to the nether portions of a horse, that noble animal for which the ancients possessed such great admiration and fondness. To Chiron was attributed many good traits and virtues. He was renowned as a hunter and famed as a musician, but most distinguished for the practice of medicine; and his teachings were such that some of his pupils became noted among the heroes of Grecian story. To the care of Chiron the infant Æsculapius was confided, who, growing up to fulfil prophecy, made an immortal name as a physician, and of whom it is even related that in one instance he restored the dead to life. When Æsculapius performed this miracle it is said that Pluto, in resentment, implored Jupiter to destroy him, which he did with a bolt of lightning, but, relenting, revived him and made him a god, placing him on a throne among the stars. Thus we see exemplified the fate which has so often befallen men of lofty achievements, wherein the honors due to true greatness are accorded only after the actors have passed away from the scenes of their beneficent labors.

Following the current annals of those early times, we find two sons of Æsculapius surgeons on the fields of the Trojan war. One of them, on the advent of peace, is reputed to have practiced venesection upon the daughter of the king of Caria, and, so the romance goes, he received her hand in marriage as his recompense. Other descendants of Æsculapius held sway as leading practitioners for five hundred years.

Next follows the brilliant record of Hippocrates, the "father of medicine." It is known that he first operated for renal calculi by incision, resorted to percussion to demonstrate the presence of fluid in the thorax, practiced paracentecis thoracis, employed the trepan, and used the obstetrical forceps.

Glancing further down the pages of medical history, the following facts are noticeable among the more remarkable contributions to our science: Praxagoras, emboldened by the hope of averting otherwise inevitable death, braved the danger of penetrating the peritoneal cavity, and made an artificial anus in a case of obstruction of the bowel of ileus. Herophilus was the first to discern that anatomy is the groundwork of medical practice, and instituted dissection of human subjects for this science as early as 320 B.C. Xenophon gave impetus to a great advancement in surgery by the invention of the tourniquet for controlling the loss of blood from the extremities. But the greatest mechanical ingenuity was displayed by Ammonicus, who devised lithotripsy, using an instrument especially invented for the purpose. Prior to this time, calculi were removed from the bladder by a special class, who made the median incision. Celsus wrote upon injuries of the head, and noticed that there could be effusions and compressions of the brain without fracture of the skull, described the hare-lip operation, and recommended the application of ligatures to arteries. A. D. 130, Galen described luxation of the femur backwards—a displacement until that time unrecognized. Ætius, A. D. 475, removed hemorrhoidal tumors and wrote on the subject of hernia. Albucasis, 1100 A. D., ventured into the trying field of intestinal surgery, and was the first to suture wounds of the intestines.

Thus there seems to have been steady progress until all advancement ceased with the general stagnation of the dark ages. But on the revival of learning and letters and the new awaking of civilization, we find the decade 1440–1450 distinguished by the discovery of printing, when the healing art took on new life, and with rapid strides developed all along the lines of true science. The widespread distribution of printed literature placed the study within the hands of such a large number of devotees that for the next 300 years discoveries and contributions to medicine became so numerous that the mention of leading particulars, even, is far beyond the scope of this paper. In later years the labors of our own American predecessors have distinguished our land as the home of some of the most notable discoveries of all time. America gave to the world the father of abdominal surgery, the father of surgical pathology, and the father of gynecology. Our countrymen conferred upon mankind the blessings of anesthesia. In obstetrical surgery America leads her mother country many years. The Americans acknowledge no superiors in the practice of medicine, nor in materia medica, and there have been recently established laboratories for pathological and bacteriological investigations that are equal to similar institutions anywhere; and in these lines of research Europe must look to guarding her laurels. Among the more prominent discoveries by Americans may be found the following: The first enucleation of the parotid gland was by a Boston surgeon in 1798. Resection for anchylosis of the hip and knee joint was first done by Barton, of Philadelphia, in 1836. Rogers, of New York, removed both superior maxillæ back as far as the pterygoid processes, in 1824. Ergot was wrested from the empyrical midwifery of England and introduced into scientific medicine by Stearns, of New York, in 1814. Peaslee, in 1854, introduced drainage in laparotomy—an innovation that has saved many hundreds of lives. The principles of Esmarch's bandage were discovered by Gross, of Philadelphia, Esmarch merely substituting rubber for cotton bandages. In 1856 Isaac E. Taylor devised the simplest method for operating for recto-vaginal fistula. In 1867 Theophilus Parvin cured a uretro-vaginal fistula in a manner exhibiting superior skill and ability. An American performed the earliest operation of supra-vaginal hysterectomy for fibroids of the uterus (in 1853), while the first successful laparotomy for pelvic abscesses was done in our land. M. B. Wright, of Cincinnati, perfected the combined method of version, which was subsequently and unjustly accredited to Braxton Hicks, of London. Storrer, of Boston, performed ablation of the uterus after Cæsarean section eight years before Porro actually performed the operation that bears his name. Washington Atlee, in 1844, did laparo-hysterotomy for fibroids of the uterus.

No section of our country has made more notable contributions to medicine than our own Southland, despite disparagements in want of hospital advantages. Boynham, of Virginia, deliberately and boldly did laparotomy for ectopic pregnancy in 1871, and again in 1879. John King, of South Carolina, performed the vaginal operation for the same condition in 1816. Kinloch, of South Carolina, was the first to practice laparotomy for gun-shot wounds of the intestines. Glover of the same State, in 1801 removed a part of the spleen protruding after a stab wound. In 1810, Deadrick, of Tennessee, did the first excision of the lower jaw. Stone, of Louisiana, made the first resection of the ribs.

But coming home to Georgia, we find the first operation for removal of lung tissue, in 1821, by Antony, and in 1819, Daniel, of Savannah, putting into practice all the principles of Buck's extension apparatus for the treatment of fractures by weights and pulleys. In 1850 the first abdominal hysterectomy for cancer of the uterus was done by Paul F. Eve; and in the same year Dr. Jeter performed laparotomy for rupture of the parturient uterus, removing the fetus and placenta from the abdominal cavity. The first total extirpation of the parotid gland was done by a Georgia doctor in the mountainous region of the State. Richard Banks, of Gainesville, performed it, the operation being so extensive that it necessitated disjointing the tempero-maxillary articulation. The elucidation of the diagnosis of yellow fever, the only certain method of diagnosing dislocation of the shoulder joint, the discovery of the excito-secretory system of nerves, and the introduction of oophorectomy, were done by physicians of this State, who were made eminent by reason of these labors. End to end anastomosis of the intestine was first successfully performed by a member of this body, whose presence graces our meeting to day. The discovery of anesthesia, and the first successful operation under its influence (1842), were incidents in the life of a village doctor of Georgia, and yet there are others who strove, and whose advocates are still striving, to obscure the just claim of Crawford W. Long as the giver of this inestimable boon to the profession and to humanity. Who is not proud to own relationship to this splendid list of eminent Georgians? Such an exhibition of surpassing genius and ability should excite the ardent admiration of every young physician in our midst, and cause him to feel that it is indeed an honor to follow in their foot-steps, and enjoy a membership in a society whose records are brilliant with their immortal names and achievements. And the pride we feel in recalling and recognizing these glorious results should move us to make an effort and see to it that the honors they deserve shall not be perpetuated alone by an occasional blot of cold type, but that their fame should have more conspicuous recognition. How fitting it would be if our legislature should erect a monument to such men as Crawford W. Long and his compeers, adorned with the record of their names and splendid labors!

May I not here suggest the reason why our Association has (as far as there has been failure) failed to impress its ideas upon public legislation? Our organization has not been so complete in form and in methods as the strength and dignity of the body would warrant. I have thought that we should appoint and sustain a standing committee on legislation, charged with the duty of thoroughly organizing the profession of the State by congressional districts and by counties for the purpose of advancing the interests of medicine, surgery and hygiene; to protect the people from the impositions and frauds of quacks and charlatans; to guard against the occurrence and spread of epidemics; to reinstate and improve the laws regulating the action of the State Board of Health, clothed with enlarged and better defined powers and duties, and to furnish earnest support and encouragement by active and helpful coöperation with the State Board of Medical Examiners. Without the coöperation and faithful support of the membership of our State Association, those to whom are committed the power and responsibility of enforcing the provisions of public statutes must be, perforce, compelled to give us but a "lame and impotent" administration of those laws, while with our active support the laws will become vitalized to work out and accomplish the best results.

Is it not opportune that our Association should take some active measures against the questionable methods in vogue, or at least of frequent occurrence, in connection with the obtainment and application of expert testimony in trials in our courts? Whenever it appears that a certain set may be found giving testimony in a common class of cases, and uniformly and constantly on the same side of the question and for the same attorneys, is it not time and occasion for the public to pause and ask if common justice is not being violated and common honesty forgotten? Is it a wonder that such circumstances bring discredit upon the whole profession, and that it is called to task for permitting such practices? Is there not a way to abolish the system by which the expert is the paid witness of either one side or the other, so as to do justice to all and remove the physician from even the appearance of being biased on the side from which he may receive pay for his time, trouble and expert knowledge? This is a question which might profitably engage the practical attention of our body, whose solution made in a wise and just spirit, backed by the active influence and indorsement of our whole membership, might crystalize into the law of the land, greatly to the promotion of the cause of truth and justice for all time to come.

Another matter worthy of our earnest attention and endeavors is the duty of urging the establishment by the State, as a branch of our Insane Asylum, a hospital for the treatment and cure of that considerable number of patients which science has demonstrated to be curable by surgical or gynecological operations. An authoritative announcement of our body, widely disseminated, that many of these unfortunate charges on the public might be restored to useful citizenship by proper treatment, and this announcement backed up by united effort on our part, would so mould public opinion that such a hospital might be speedily provided—an institution that in the end would effect a saving in the public expense and greatly promote the cause of humanity. Surely it is the part of our Association to make the need known, and to rest not until there is established this true blessing to the State. Without suggesting further details, it might seem that such an institution should be located at some railway center, so as to be readily accessible, and its work placed in charge of a surgeon and gynecologist, together with an alienist and a nerve specialist, in order that the highest and best results be obtained.

Congratulating our noble board of censors who have so efficiently performed the duties—the delicate duties—of their trust, let us uphold their just actions. Theirs indeed is a difficult task. They must ascertain and decide upon acts and facts in the conduct of brothers that constitute a breach of our professional rules of conduct, the safeguard of our honor and our real welfare. Far removed from every personal consideration, with the deepest desire to discover the truth and do the right, uncaring consequences, but approaching their task with brotherly charity, it is their office to give gentle admonition of breaches of minor rules, but fearlessly to report facts when grave infractions occur. Let us give our censors thanks and support in their arduous work. It is theirs to note, not in harshness, the failing and mischances of our erring brothers, with a view to friendly correction; it is for us to stamp the seal of condemnation when deserved, and lifting still higher the spotless banner of true professional ethics, find delight to battle under its folds to the end.

These few suggestions, gentlemen, are but the outlines of the picture of public beneficence it is in the power of the Medical Association of Georgia to frame for the admiring view of our people, if we will but come close together with new and firm resolve to act in vigorous concert, and with true professional and personal zeal.

Let us here, amid the pleasant social surroundings of our annual meeting, where we have for a season turned our backs on the phantoms of sickness and suffering and sorrow, renew again and strengthen our pledges of devotion to our profession.

